# Supplementary material for: Benchmarking of shotgun sequencing depth reveals the potential and limitations of shallow metagenomics and strain-level analysis
Source: Nat Microbiol. 2026 Apr 21;11(5):1233–44. doi: 10.1038/s41564-026-02334-2 (PMC13171431; doi:10.1038/s41564-026-02334-2)
Supplement: Supplementary file 1 — Supplementary Figs. 1–5: relative abundance per reference genome or after ‘non-supervised’ taxonomic assignment using MetaPhlAn4 for Mock-even-70, Mock-stag-24 and Mock-stag-70. [file 41564_2026_2334_MOESM1_ESM.pdf]

nature microbiology

Supplementary information

https://doi.org/10.1038/s41564-026-02334-2

Benchmarking of shotgun sequencing depth reveals the potential and limitations of shallow metagenomics and strain-level analysis

In the format provided by the authors and unedited

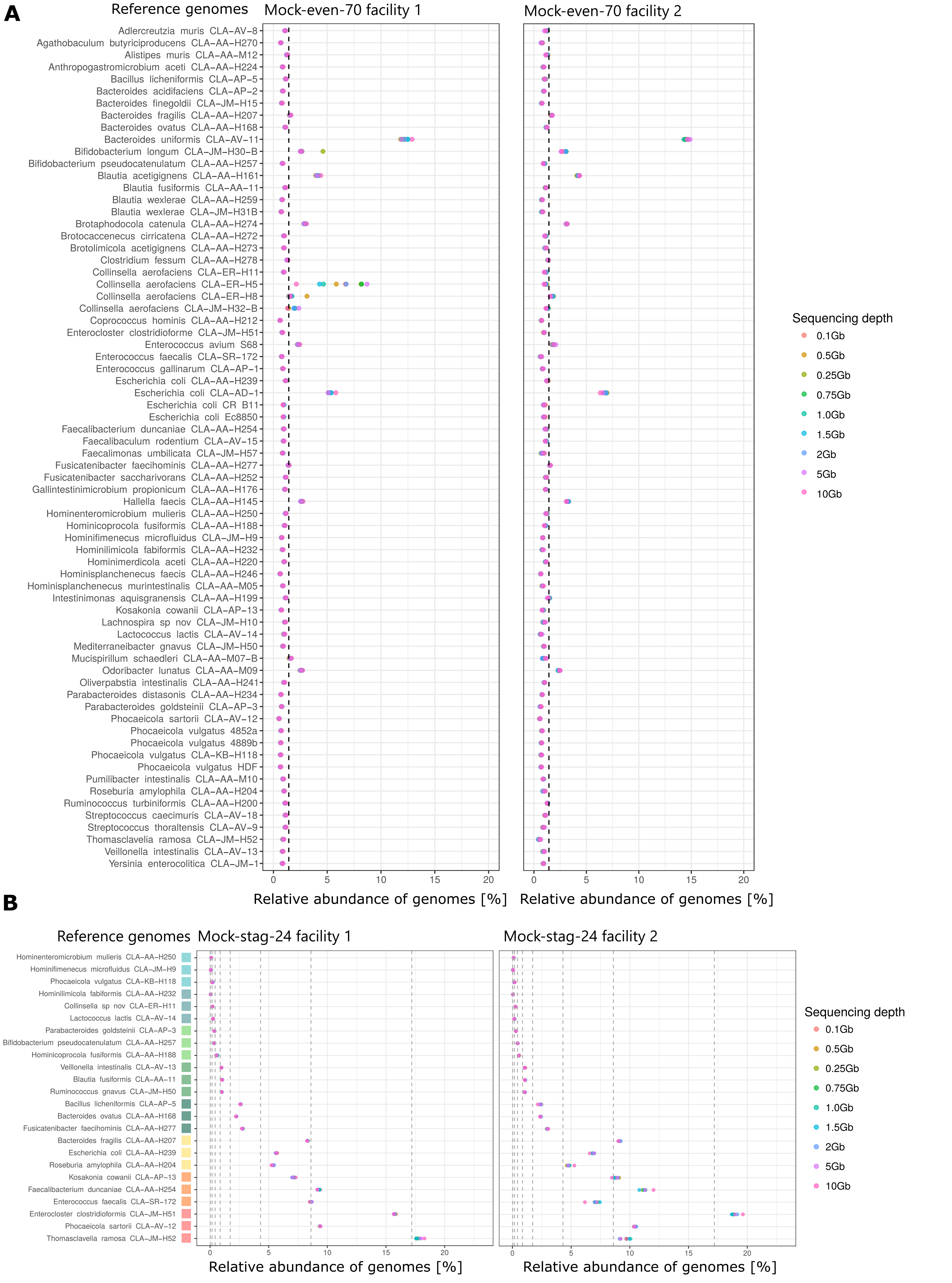

Supplementary Fig. S1: Relative abundance per reference genome
Relative abundances based on reference genomes in (**A**) Mock-even-70 in facility 1 and 2, (**B**) Mock-stag-24 in facility 1 and 2. Sequencing depths are indicated by the coloured dots. The theoretical relative abundances are indicated by vertical dashed lines. For Mock-stag-24 in panel B, the reference genomes are ranked from top to bottom by increasing DNA amount in the mixture indicated by coloured boxes (from blue to red; 0.04, 0.4, 1, 2, 4, 10, 20, 40 ng)(concentrations are provided in **Supplementary Data S1**).

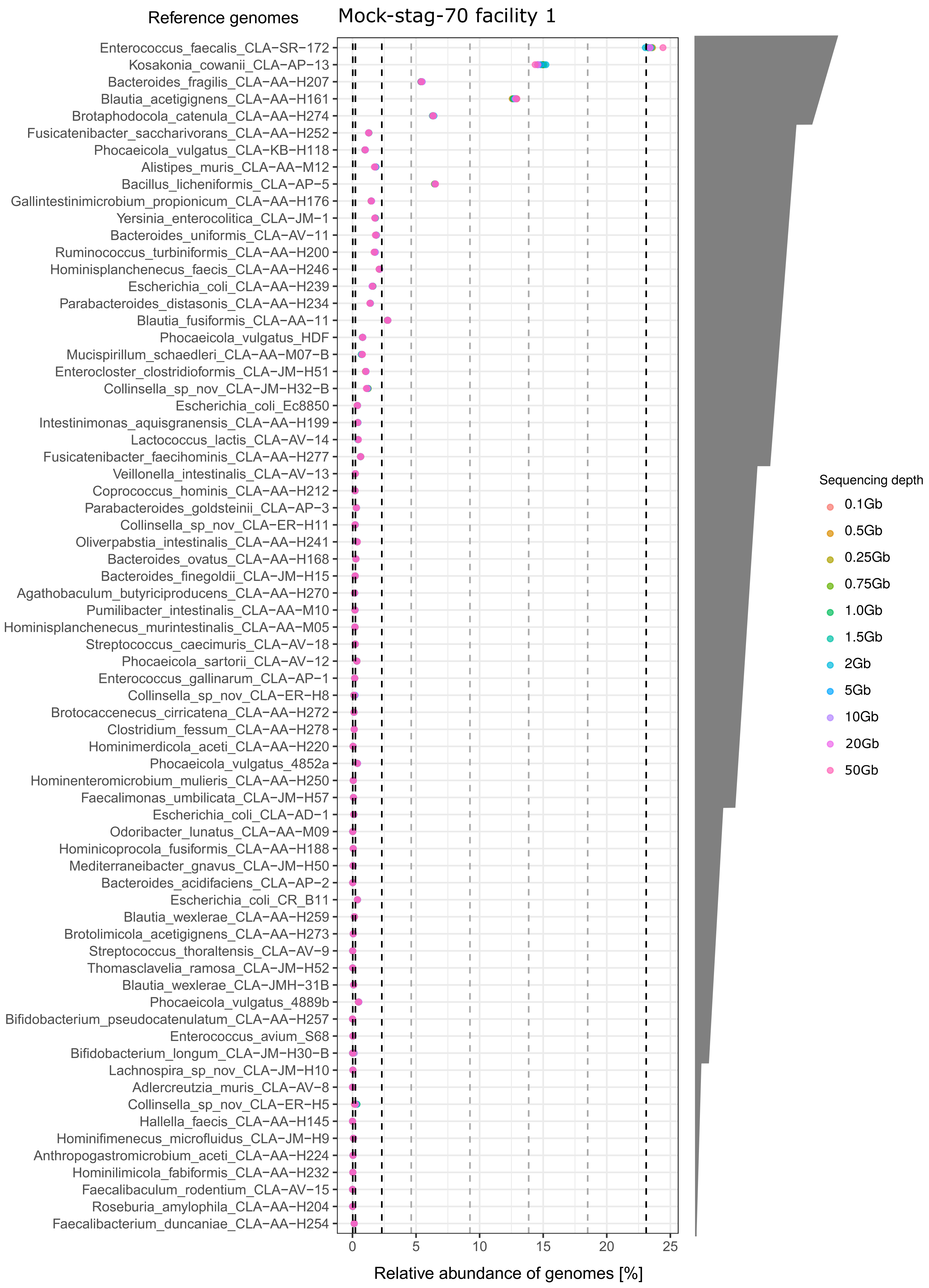

Supplementary Fig. S2: Relative abundance per reference genome
Relative abundances based on reference genomes in Mock-stag-70 in facility 1.
Sequencing depths are indicated by the coloured dots. The theoretical relative abundances are indicated by vertical dashed lines. The reference genomes are ranked from top to bottom by decreasing DNA amount in the mixture indicated by the grey gradient (concentrations are provided in **Supplementary Data S1**).

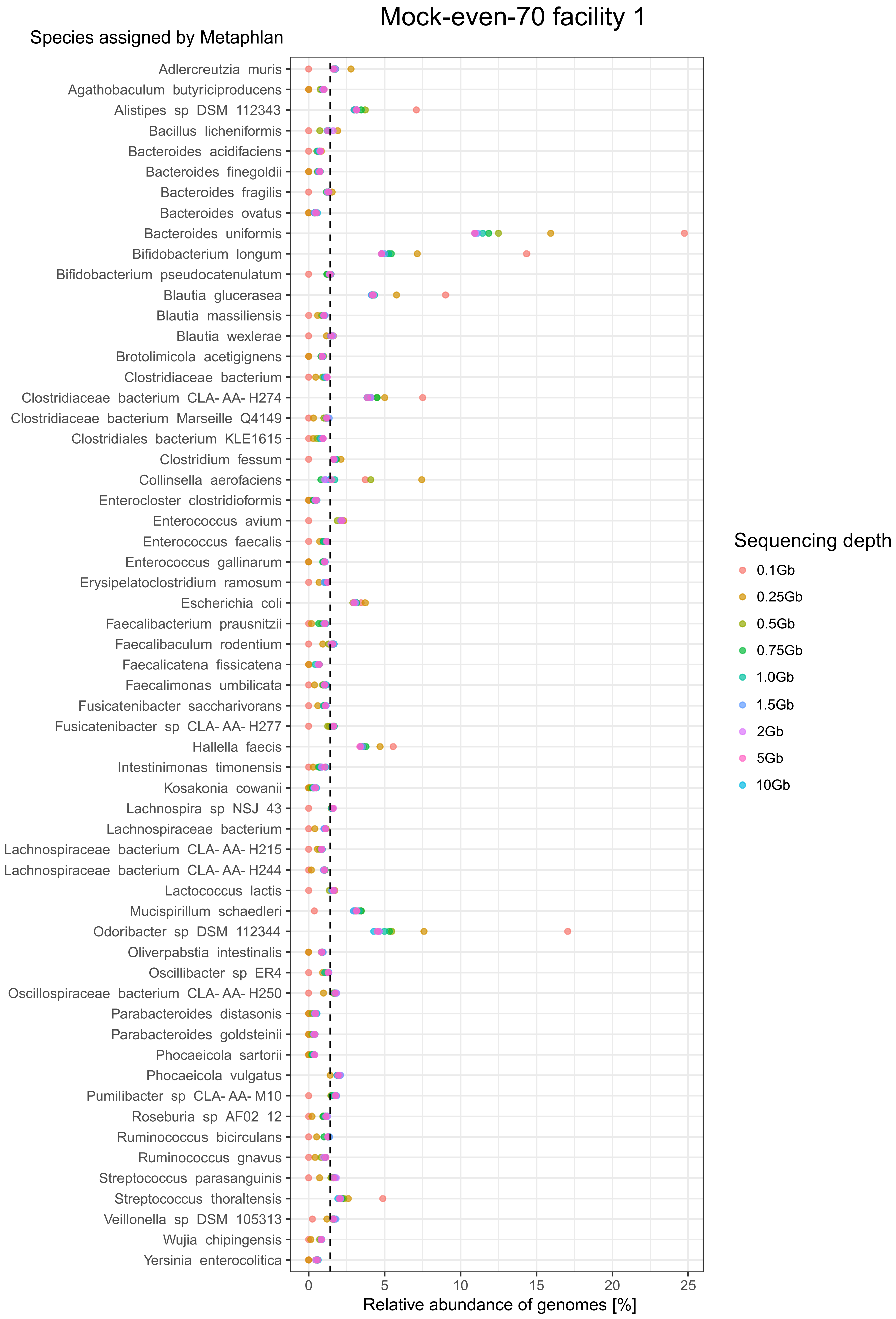

Supplementary Fig. S3: Relative abundance after "non-supervised" taxonomic assignment using MetaPhlAn4 in Mock-even-70. Sequencing depths are indicated by the coloured dots. The theoretical relative abundance is indicated by a vertical dashed line.

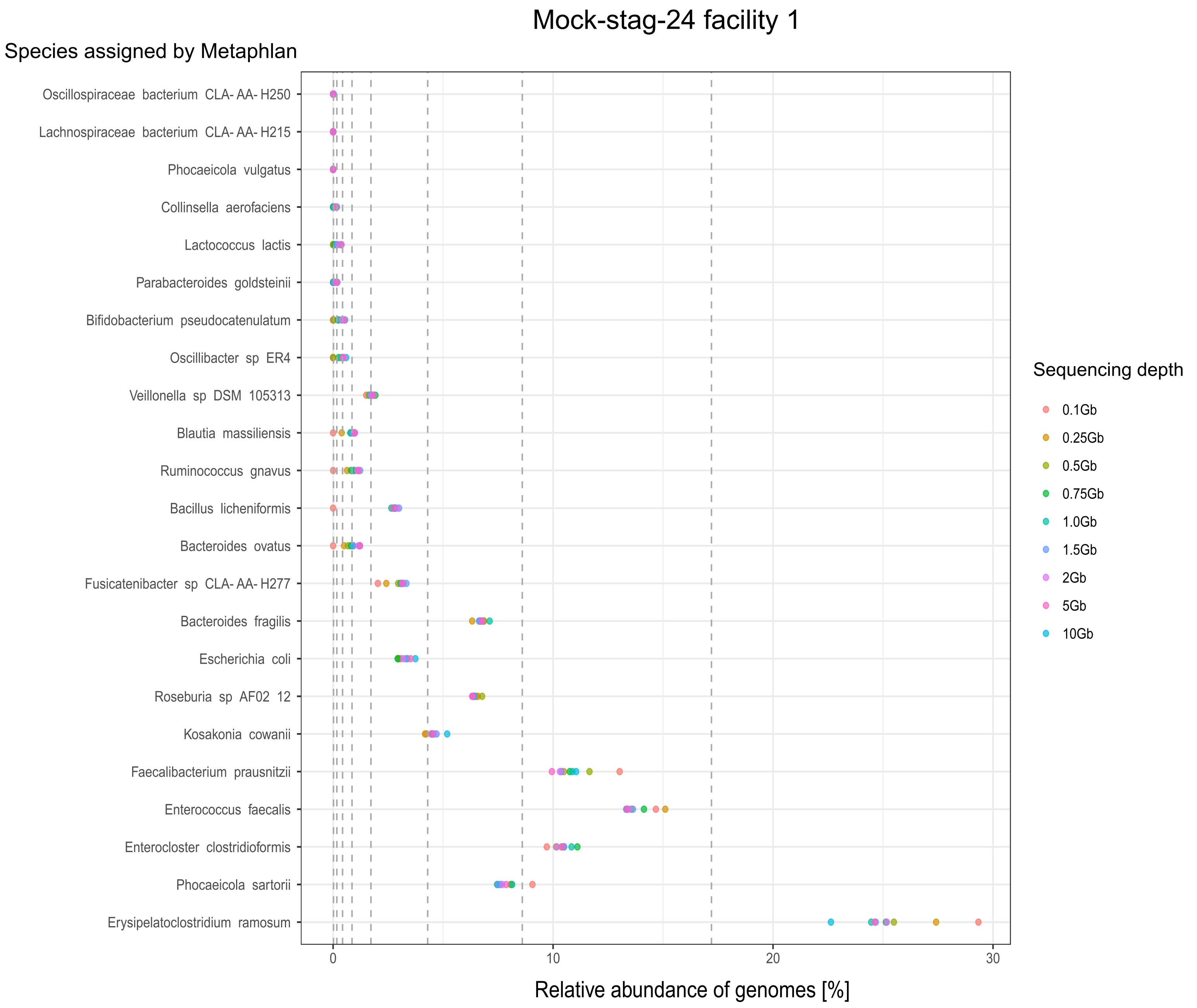

Supplementary Fig. S4: Relative abundance after “non-supervised” taxonomic assignment using MetaPhlAn4 in Mock-stag-24. Sequencing depths are indicated by the coloured dots. The theoretical relative abundances are indicated by vertical dashed lines.

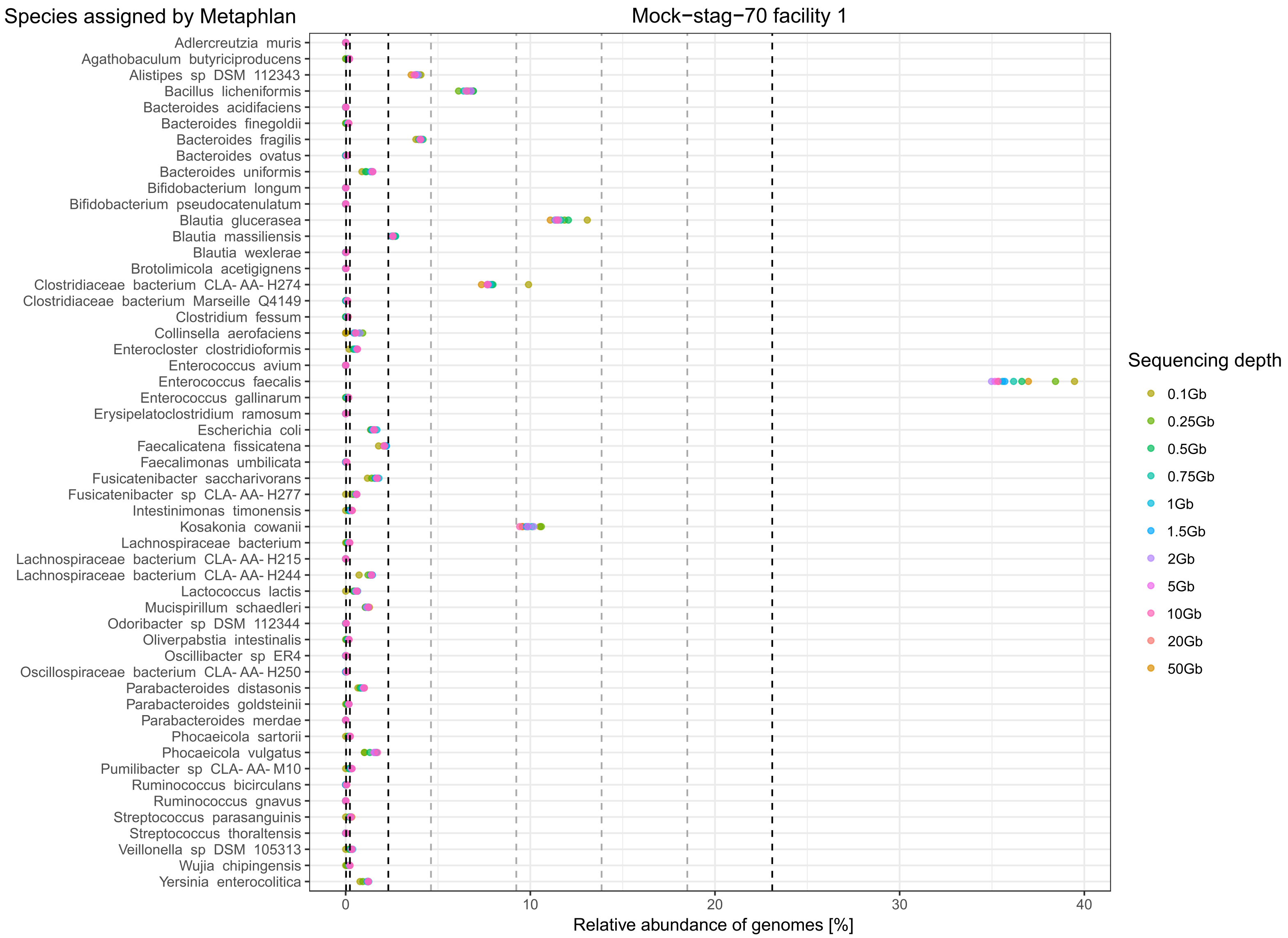

Supplementary Fig. S5: Relative abundance after “non-supervised” taxonomic assignment using MetaPhlAn4 in Mock-stag-70. Sequencing depths are indicated by the coloured dots. The theoretical relative abundances are indicated by vertical dashed lines.
